# Quantifying animal social behaviour with ecological field methods

**DOI:** 10.1098/rsos.241299

**Published:** 2025-01-15

**Authors:** Molly A. Clark, Christos C. Ioannou

**Affiliations:** ^1^ School of Biological Sciences, University of Bristol, Bristol, UK; ^2^ Department of Biological Sciences, Macquarie University, Sydney, Australia

**Keywords:** aggregation, assortment, collective behaviour, environmental stressors, *Gasterosteus aculeatus*, index of dispersion

## Abstract

Field studies of social behaviour are challenging due to the need to record or infer interactions between multiple individuals, often under suboptimal environmental conditions or with potential disturbance by observers. Due to the limited field techniques available, we present a novel method to quantify social behaviours in the field by comparing the counts of individuals caught in traps across multiple locations sampled simultaneously. The distribution of individuals between traps gives the extent of aggregation, and phenotypic data allow for inference of non-random assortment. As a case study, we applied this method to populations of three-spined sticklebacks (*Gasterosteus aculeatus*) in freshwater ponds, using minnow traps. As expected, we observed a strong trend for aggregation. We were able to describe the ecological drivers of aggregation, comparing environmental and phenotypic conditions across sites. Aggregation was not related to environmental parameters, but was negatively associated with the proportion of breeding males caught during the breeding season. No evidence for phenotypic assortment based on body size was found. These results demonstrate that widely available ecological equipment can address questions related to social behaviour. This cost-effective approach, avoiding the tagging of individuals and minimizing extended observer disturbance, can be applied across various habitats and species.

## 1. Introduction

Social behaviour is essential for the survival and reproduction of many species: being in a group provides energetic [1], reproductive [2], foraging [3] and anti-predator benefits [4], but can also incur costs [5,6]. These costs and benefits associated with grouping can change depending on environmental conditions, altering social organization within a population through adaptive changes in individuals’ behaviour [7,8]. Environmental conditions can also interfere with and constrain the inter-individual interactions that allow for group formation and maintenance [9,10]. Because of widespread environmental change due to human activity, it is increasingly important to study the effect of environmental factors on social behaviour so we can understand changing dynamics in wild populations [8].

Methods for studying social behaviour in the field typically depend on *in situ* observations [11,12], video recordings [13,14] or technology such as Global Positioning System and Radio Frequency Identification tags attached to individuals [15,16]. Only a small proportion of habitats are suitable for direct data collection by observers or cameras [17] and require a substantial investment of observers’ time. Further, direct observation methods are only reliable for species that can habituate to the presence of observers. Biologging tags can be programmed to automatically collect spatial and other data, although to reliably measure social behaviour (typically inferred from the proximity of individuals) requires data to be collected from a large proportion of the population to avoid missing social interactions; thus, this approach involves extensive handling of animals and high financial investment. Tags can also be invasive with implications for welfare, and it is unclear to what extent they affect the natural behaviours of tagged individuals [18,19].

Both direct observation and tagging are limited in aquatic habitats, where video and sonar are more commonly used [20,21]. While action cameras have made filming much more cost effective, videoing underwater remains highly sensitive to environmental conditions and hence has limited applicability [22,23]. Further, these restrictions are often caused by the conditions we want to study, such as variation in light intensity, turbidity or habitat complexity from vegetation, which are of critical importance as freshwater ecosystems are among the most biodiverse yet vulnerable to anthropogenic disturbance [24,25]. Sonar imaging allows recording in the field when there is poor visual acuity [26,27]; however, it has limited spatial and temporal resolutions, and species identification can be difficult [27].

**Figure 1.**
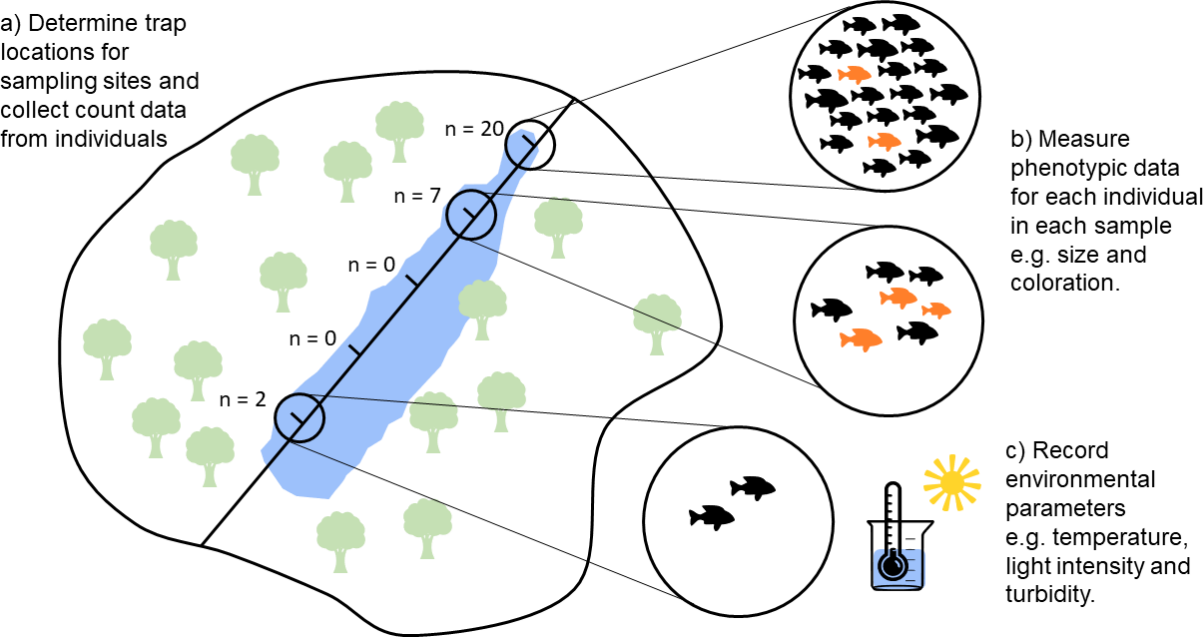
Schematic of our method and the three components (*a*), (*b*) and (*c*).

Considering the restrictions of current techniques, we have developed a method that aims to quantify social behaviour in the field that involves counting the number of individuals caught in traps that are deployed simultaneously at multiple locations within a limited area, for example, along a transect. The trapping method needs to be relevant to the species, such as minnow traps for small fish, pitfall traps for insects, or camera traps for birds and mammals (figure 1*a*). The distribution of individuals between the traps deployed in a sampling session provides a measure of how aggregated those individuals are; for example, if all individuals observed are found in a single trap, that implies a high degree of aggregation. Aggregation can occur through individuals being together in a group and entering the trap together; alternatively, aggregation can occur through individuals being attracted to those already caught in the trap. These can be distinguished when trap design facilitates the recording of trapping occurrences, for example, with camera traps [28]. Regardless, the approach measures social tendencies, i.e. the degree to which individuals associate with other individuals [29], of the population. Further questions relating to social behaviour, such as whether particular phenotypes are correlated with stronger social tendencies [29], can be addressed by collecting data on the phenotypes of individuals caught in the traps, for example, species, size, colouration and parasite load (figure 1*b*). Collection of environmental data before, during and/or after the sampling session at each site, e.g. temperature and light intensity (figure 1*c*), can be used to test for the association between social behaviour and environmental parameters [7].

Here, we apply this method to three-spined sticklebacks (*Gasterosteus aculeatus*) in freshwater pond habitats as a model system, using the common fishing gear passive funnel traps (a.k.a. minnow traps) as traps (figure 1*a*, electronic supplementary material figure S1). We demonstrate how the data provided by this approach can be analysed statistically to address hypotheses related to aggregation, phenotypic assortment and their association with abiotic variables. We used sticklebacks due to their prevalent use in behavioural research and because they are commonly found in fresh and brackish water in the UK. Sticklebacks are facultatively social and have been found to prefer to join groups of individuals similar to them in size [30], thus we predicted we would observe non-random phenotypic assortment in body size. Of particular interest was how environmental parameters are associated with social behaviour [7]. From the previous literature, we would expect fish to be more aggregated (i.e. less evenly distributed across traps) at lower temperatures ([31], although see [32]), while being less aggregated in low oxygen conditions [33]. Visual constraints could also lead to fish being less aggregated when turbidity is higher [9,34] and where there is lower light intensity [35], due to vision being an important sensory modality for shoal cohesion [36].

## 2. Material and methods

### 2.1. Study sites

The study was carried out in four ponds in Bristol, UK (table 1). Pond sites were chosen based on having adequately large populations of three-spined sticklebacks to catch enough fish per sampling session for analysis of aggregation and assortment by body size. Site choice was also dependent on accessibility and appropriate water depth for traps to be deployed. Sampling carried out at these sites was approved by the Environment Agency UK.

**Table 1.** Mean ± standard deviation of environmental variables recorded at each pond site. These are calculated by averaging the recordings from each trap location per sampling session and then averaging these session means over the 13 weeks of data collection, which took place from May to November 2021.

parameter	Brandon	Pennywell	Sneed	Tarn
National grid reference	ST57967293	ST55737783	ST55477549	ST55887818
temperature (^°^C)	16.29 ± 4.40	13.44 ± 2.75	16.50 ± 4.03	14.68 ± 3.80
turbidity (NTU)	2.72 ± 1.48	13.09 ± 8.17	5.25 ± 1.88	36.43 ± 14.41
dissolved O_2_ (mg l^−1^)	11.35 ± 1.95	9.21 ± 1.19	8.63 ± 2.25	9.06 ± 1.77
light intensity (lum ft^−2^)	631.49 ± 445.64	191.15 ± 171.65	790.83 ± 644.35	476.83 ± 265.34
mean ± s.d. catch per site	85.38 ± 69.66	101.62 ± 77.25	117.38 ± 108.77	66.23 ± 52.63
mean ± s.d. catch per trap	14.68 ± 19.67	21.92 ± 33.11	20.72 ± 30.29	17.26 ± 28.91

## 3. Experimental procedure

**Figure 2.**
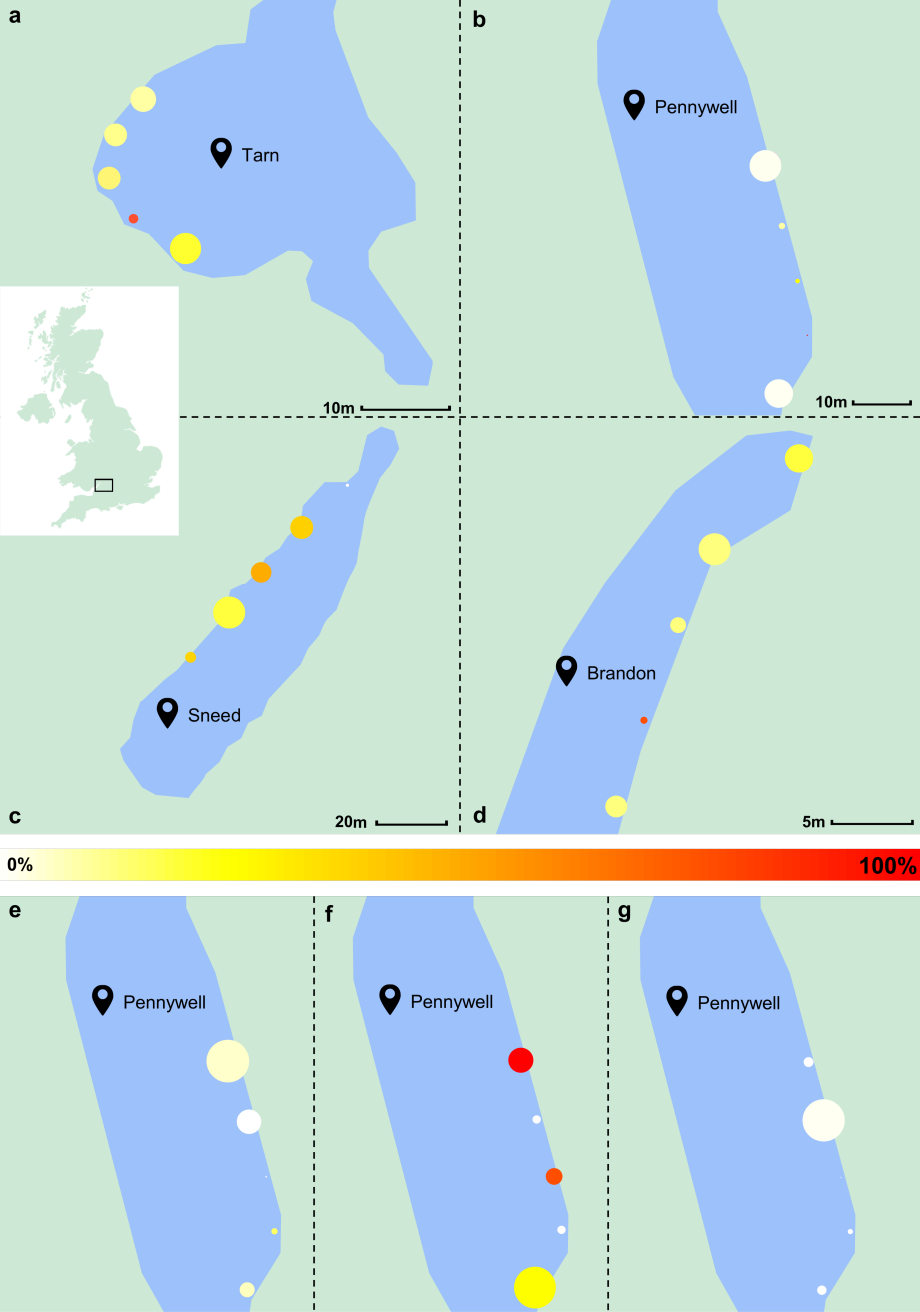
(*a–d*) Maps of each pond site, showing data from week 3 of collection. Points indicate trap locations (1–5). (*a*) Tarn: mean trap distance = 4.7 m, area ~ 0.075 ha; (*b*) Pennywell: 6.7 m, approximately 0.16 ha; (*c*) Sneed: 8.8 m, approximately 0.25 ha; (*d*) Brandon: 4.9 m, approximately 0.016 ha. (*e–g*) Pennywell in weeks 4 (*e*), 5 (*f*) and 6 (*g*). Trap locations (1–5) are shown, with point size scaled to the number of fish caught (max size = 5) and colour representing the proportion of red breeding-condition males (0% = white, 100% = red). Number of fish caught across the pond site (*n*) and aggregation score: (*a*) *n* = 122, aggregation = 8; (*b*) *n* = 181, aggregation = 39; (*c*) *n* = 196, aggregation = 35; (*d*) *n* = 104, aggregation = 12; (*e*) *n* = 114, aggregation = 19; (*f*) *n* = 12, aggregation = 1; (*g*) *n* = 130, aggregation = 38. Images adapted from Google Maps.

The study was designed to quantify the aggregation in the population by comparing the numbers of fish caught across five funnel traps simultaneously deployed during a sampling session in a pond site (electronic supplementary material, figure S1). Traps were deployed at equidistant locations along a pond, as much as access and water depth allowed (figure 2); these trap locations were maintained throughout data collection and labelled 1–5 from left to right (see points on figure 2). Traps were either dropped slowly into the water using the long string attached to the trap (electronic supplementary material, figure S1) where it was possible to get close enough to the trap location, or thrown by holding two corners of the trap when the trap needed to reach a further distance, for instances where water was too shallow near the bank or there was poor access to the edge of the water. Deployment required careful positioning to ensure the temperature and light intensity logger (HOBO MX2202) attached to each trap was facing upwards in the water, and misalignment would require pulling the trap back in with the string and redeploying it in the same location. Traps were baited in the first week with bread, but tended to attract other species such as carp; therefore, traps were not baited for subsequent trials to avoid attracting unwanted species. After the two-hour sampling period where traps were left in the water undisturbed, they were pulled in using the string attached to each trap. If the trap contained fish, it was quickly moved to an area of shallow water in the pond so that all caught fish were fully submerged, but the water was shallow enough to prevent escape through the openings in the trap. This allowed time for counting and measuring the fish while preventing unnecessary stress.

Sampling took place on alternating weeks between May and November 2021. Which pond site was sampled on which day during a week of data collection was decided by random shuffling of the sites in R v. 3.3.3 [37]. Traps were left for 2 h between either 10.00 and 12.00 or 13.00 and 15.00 to account for any confounding effect of time of day. The first sampling time for each site was chosen by coin flip and alternated between am and pm thereafter for each site.

### 3.1. Environmental variables

Water temperature and light intensity were recorded using a HOBO MX2202 attached to each trap, which logged data every minute for the two-hour sampling period. Turbidity and dissolved oxygen were measured from water samples taken at each trap location, with samples taken before and after the traps were deployed (i.e. at the start and end of each sampling session). Turbidity was measured using a Thermo Scientific Orion AQUAfast AQ3010 Turbidity Meter and dissolved oxygen using a Lutron Dissolved Oxygen Meter PDO-519.

### 3.2. Fish measurements

Fish caught in each trap were counted, also noting the number of males in breeding condition with characteristic red colouration [38]. Body length measurements were taken by placing groups of fish from the same trap in a bucket (electronic supplementary material, figure S2) with a 10 cm scale bar and water from the pond to a depth of 5 cm. The number of fish in the bucket varied between 1 and 21, depending on the number of fish caught in the traps and their size (a greater number of juveniles could be imaged accurately). A GoPro Hero5 was attached to the side of the bucket, 27 cm above the water surface and oriented downwards to give an overhead view of the fish. An image of the appropriate trap number (1–5) was taken before photographing the fish from each trap in the bucket to ensure all images were correctly attributed to the corresponding trap. Images of 4000 × 3000 pixel resolution were taken in burst mode, where 10 photos were taken over 2 s; this was repeated multiple times if fish were closely aggregated, with the aim to capture an image where all fish were clearly visible and not overlapping. After photographs were taken, fish were released from the bucket back into the pond. This was repeated for all fish caught in a trap before processing the next trap. Body length measurements were then made using ImageJ (v. 1.53; 39). This procedure allowed for reduced stress to the fish and more efficient data collection than manual handling and measuring each fish individually, for example, using callipers.

## 4. Statistical analysis

All analyses were conducted in R (v. 4.1.2 [37]). The number of fish caught per trap in a sampling session, the proportion of adults caught per trap in a sampling session and the aggregation of fish across the traps in a sampling session were analysed as response variables in separate generalized linear mixed-effect models (GLMMs). All GLMMs were run using the glmmTMB function (*glmmTMB* [40]). The assumptions of all models were verified using QQ plots and dispersion tests using the residual diagnostics for mixed regression models (DHARMa [41]). GLMMs for each response variable were run with a single fixed effect (e.g. temperature) and random effect (either pond site or trap location nested in pond site, depending on the response variable). Models with only one fixed effect were used based on the consideration that including multiple fixed effects in GLMMs can substantially reduce statistical power, especially in combination with small sample sizes [42].

The mean of the temperature and light intensity recordings made from the start to the end of the sampling period was calculated to give an average temperature and light intensity value for each trap location for every sampling session. Turbidity and dissolved oxygen measurements from the start and end of each sampling session, from each trap location, were also averaged (mean). When analysing response variables that only had a single value per sampling session (i.e. aggregation score), rather than at each trap location (e.g. total fish caught per trap), the environmental variables were averaged (mean) across the five trap locations. To test for correlations between the continuous explanatory variables (i.e. temperature, light intensity, dissolved oxygen, turbidity and week of data collection), relationships were tested with Spearman’s rank correlation coefficient (*r*_s_) in R [43]. A Friedman test was conducted separately for each pond site to determine whether the fish had a preference for particular trap locations within a pond site by determining whether the number of fish caught differed significantly across trap locations within the pond sites. The Friedman test, a non-parametric method, was selected because it accommodates the repeated measures design with data collected from the same trap locations over multiple weeks. The statistical significance was determined using *p*-values, with *p* < 0.05 indicating a significant difference in the number of fish caught across trap locations, and therefore demonstrating a preference for particular trap locations within a pond site.

### 4.1. Total fish caught

Although our method is designed to quantify social behaviour, the total number of individuals caught may also be of interest, either independently of social behaviour or as a variable that is potentially associated with social behaviour. To determine which variables could explain the variation in the number of fish caught per trap per sampling session, we compared seven GLMMs with a negative binomial distribution and the default log link function. The response variable was the total number of fish caught per trap, and trap location nested within pond site was included as the random effect. Each of the seven models had a different fixed effect: temperature, light intensity, dissolved oxygen concentration, turbidity, the week of data collection (1 to 13), and whether sampling occurred in the morning or afternoon (am or pm); a null model with no fixed effect was also included in the model comparison set. We then compared the Akaike information criterion (AICc) values corrected for small sample sizes for each model using the ICtab function in R (*bbmle* v. 1.0.24 [44]). Models with lower AICc values are more likely given the data, and the model with an ΔAICc of zero is the most likely model. Explanatory variables included in models that had AICc values of greater than two units less than the null model were considered to be strongly supported as important predictors of the response variable [42]. In addition to comparison to the null model, we could also determine which explanatory variables were more likely to predict the variation in the response variable than others. The slope (coefficient), standard error of the coefficient and incidence rate ratio (IRR) were calculated for each model to provide more biological context to the relationship between the response variable and fixed effects, i.e. the direction of the relationship, the reliability of the estimate and an interpretable measure of the effect size, respectively.

### 4.2. Proportion of adults and juveniles

As with the number of fish caught, our method allows the phenotypes of the population to be quantified, as well as their social behaviour. Phenotypic characteristics we recorded were the standard body lengths of all individuals and whether individuals displayed red colouration characteristic of male sticklebacks in breeding condition [38]. Visualization of the body length data revealed a clear divide between adults and juveniles when plotted over the weeks of data collection (i.e. over time; electronic supplementary material, figure S3). We clustered the fish into two groups, adults and juveniles, based on this trend, where adults refer to fish large enough to be caught from the start of the data collection in May, while juveniles were those not large enough to be caught at the start. The trends in electronic supplementary material, figure S3 are consistent with the numbers of adults and juveniles we would expect to catch over time as a result of the progression of the spawning season. To analyse the proportion of fish caught that were adults rather than juveniles, the number of adults and juveniles in each trap were combined into a two-column matrix using the cbind function in R (e.g. cbind (number of adults, number of juveniles)). This structure allows for binomial modelling of the proportion of adults among the total catch. This was used as the response variable in seven binomial GLMMs to determine which variables could explain the variation in the proportion of adults (relative to juveniles) caught per trap location. Each model included a different explanatory variable: temperature, light intensity, dissolved oxygen concentration, turbidity and morning or afternoon. The week of data collection was included as a covariate in all models because it was used to determine whether caught fish were adults or juveniles (electronic supplementary material, figure S3). A model with only week as a fixed effect and a model with no fixed effects were included as null models for comparison. Trap location nested within pond site was included in all models as the random effect. AICc values were then compared, and the coefficient, standard error of the coefficient and IRR were calculated as previously described.

### 4.3. Aggregation

**Figure 3.**
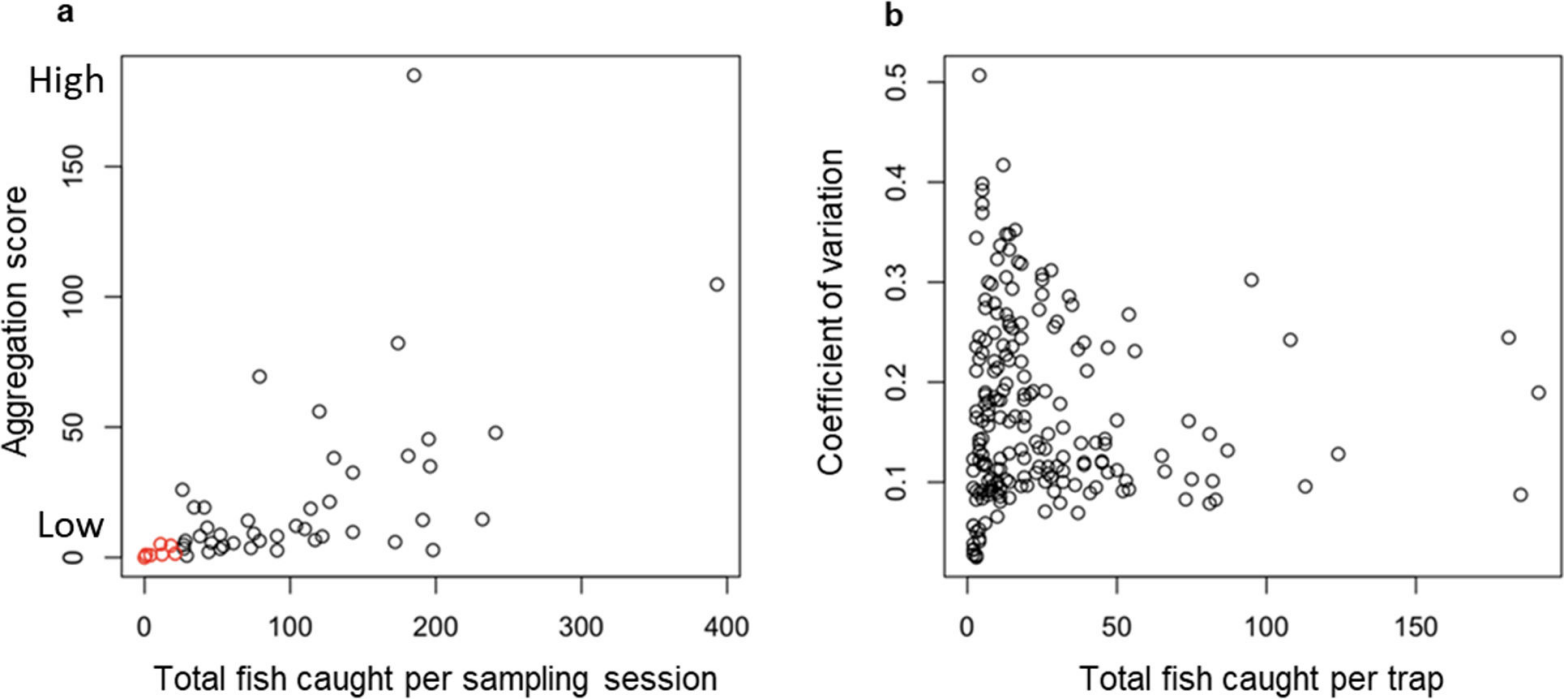
(*a*) Aggregation scores (index of dispersion, i.e. variance/mean) for each sampling session as a function of the total number of fish caught during that sampling session. Larger values on the *y*-axis represent cases where fish are more aggregated. Red points indicate data that were removed from the analysis, as the aggregation score did not seem to be able to take large values when the total number of fish caught was less than 26. (*b*) Coefficient of variation (COV) values for each trap compared to the number of fish caught in that trap. Larger values on the *y*-axis indicate higher variation in body length among fish in a trap relative to their mean body length.

The aggregation of fish among the traps for each sampling session was determined by calculating the index of dispersion (i.e. the variance/mean) from the number of fish caught in each trap to produce an aggregation score for the sampling session [45]. An aggregation score of 0 occurs when fish are evenly distributed across the traps (i.e. the same number of fish are caught in each trap) and indicates that fish are avoiding one another. A value of 1 occurs when fish are randomly distributed between the traps, and higher values indicate fish are aggregated (figure 3*a*). Cases were removed from the analysis when the total number of fish caught in that session was too low to reliably detect aggregation patterns by this measure (e.g. figure 2*f*). With a small sample size, the aggregation score (index of dispersion) can become disproportionately low because the variance and mean are constrained by the limited data. A threshold of 26 fish was established as the minimum number of fish caught to accurately reflect aggregation; the threshold was determined from plotting the aggregation score as a function of the total number of fish caught and identifying the lowest catch size where clear aggregation patterns were observed (figure 3*a*). To determine which variables were likely to predict the aggregation of fish, we compared eight negative binomial GLMMs. The response variable was the aggregation score for each sampling session, and each model had a different explanatory variable: temperature, light intensity, dissolved oxygen concentration, turbidity, week of data collection and morning or afternoon. Here, an additional model was considered, which had the proportion of red-bellied breeding-condition males caught (i.e. number of red males/total fish caught) as an explanatory variable. The eighth model was the null model that lacked an explanatory variable. Pond site was included as the random effect in all models. AICc values were then compared, and the coefficient, standard error of the coefficient and IRR were calculated as previously described.

### 4.4. Phenotypic assortment

As a measure of phenotypic assortment, we used the coefficient of variation (COV; i.e. standard deviation ÷ mean) of the standard body lengths recorded for each individual fish caught in a trap, excluding cases where only one or no fish were caught (figure 3*b* [46]). A COV of zero would indicate there is no variation in size between the fish caught in a trap; the higher a COV value, the more variation (relative to the mean body size) there is between fish caught in a trap. The median COV of all the traps from one sampling session was used as the observed COV for that sampling session at that pond site (observed siteCOV).

To determine whether fish caught in the traps were more or less phenotypically assorted by body length than expected by chance, we calculated the expected median COV for each sampling session, assuming random assortment between traps. The expected value for assortment was calculated using a constrained randomization procedure in which individuals caught across traps from one sampling session were randomly redistributed across the five traps, maintaining the number of fish caught per trap as in the observed data. The COV of body length was calculated per trap from each resampling, and the median of these values was saved as the value of expected assortment (expected siteCOV). This was iterated 10 000 times for each sampling session at each pond site and an expected distribution of assortment values was generated.

The observed siteCOV was then compared to this expected distribution. Specifically, we determined where the observed siteCOV fell within the distribution of expected siteCOV values by calculating the observed siteCOV’s quantile. To calculate the quantile, we identified the proportion of expected values that were less than or equal to the observed siteCOV. For example, if the observed siteCOV was at the 0.5 quantile, it would mean that 50% of the expected siteCOV values were less than or equal to the observed value. In two-tailed tests, quantiles are statistically significant when less than 0.025, indicating positive assortment (i.e. fish are assorting with those that are similar to themselves), or when greater than 0.975, indicating negative assortment (i.e. fish are assorting to those that are different from themselves).

## 5. Results

### 5.1. Correlation between explanatory variables

Correlations between the continuous explanatory variables included in the models showed strong evidence of correlation between temperature and light intensity (electronic supplementary material, figure S4; Spearman’s rank correlation coefficient: *r_s_* = 0.53, *p* < 0.0001, *n* = 233), week and light intensity (*r_s_* = −0.57, *p* < 0.0001, *n* = 233) and week and temperature (*r_s_* = −0.47, *p* < 0.0001, *n* = 233).

### 5.2. Differences between trap sites (Friedman test)

Analysis of the difference between the number of fish caught across the trap locations for each pond site showed that at the Brandon pond site, there was no significant difference in the number of fish caught across the trap locations (table 2). This result suggests that fish are not showing a preference for a particular site location. At Sneed, Pennywell and Tarn, the Friedman test returned *p*-values around the 0.05 threshold for statistical significance (table 2), providing some evidence that fish are more likely to be caught at specific trap locations. A portion of these patterns is visually illustrated in figure 2*e*–g.

**Table 2.** Results of the Friedman test for differences in the number of fish caught across different trap locations within each pond site. The Friedman test statistic (*X*^2^), degrees of freedom (d.f.) and *p*-value are provided for each pond site.

site location	Friedman’s *X*^2^	d.f.	*p*‐value
Brandon	2.66	4	0.616
Sneed	8.37	4	0.079
Pennywell	10.48	4	0.033
Tarn	10.13	4	0.038

### 5.3. Fish caught per trap

Analysis of the number of fish caught per trap showed that the model with week as the explanatory variable was the most likely model given the data (electronic supplementary material, table S1). The number of fish caught decreased over consecutive weeks of data collection (electronic supplementary material, figure S5a). The model with dissolved oxygen as the explanatory variable was also strongly supported compared to the null model (electronic supplementary material, table S1). More fish were caught when the concentration of dissolved oxygen in the water was lower (electronic supplementary material, figure S5b). The model with temperature as the explanatory variable was less well supported, but being greater than 2 AICc units less than the null model still provides strong evidence that temperature was associated with the number of fish caught (electronic supplementary material, table S1). In this case, the number of fish caught increased in warmer water (electronic supplementary material, figure S5c). There was also evidence that light intensity was positively associated with the number of fish caught in a trap (electronic supplementary material, figure S5d). Based on the AICc being higher than the null model, there was no evidence for turbidity or time of data collection having an effect on the number of fish caught at a trap location.

### 5.4. Proportion of adults caught per trap

Analysis of the proportion of fish caught that were adults found that the model including temperature and week was the most likely given the data (electronic supplementary material, table S2). The proportion of adults caught decreased as temperature increased (electronic supplementary material, figure S6a). The model with the time of sampling (am or pm) as an explanatory variable also had strong support, where more adults were caught in the afternoon (electronic supplementary material, figure S6b). Light intensity had similar support to the time of sampling (0.7 AICc greater than am and pm), where a higher proportion of adults were caught at higher light intensity levels (electronic supplementary material, figure S6c). The model with turbidity as the explanatory variable was less well supported than other models but had an AICc of 6.1 less than the null, thus still having strong support. Here, there was a positive relationship, where the proportion of adults caught was higher in more turbid conditions (electronic supplementary material, figure S6d). The model with dissolved oxygen as an explanatory variable was within 2 AICc of the model including only week, therefore, there was no strong evidence that including dissolved oxygen in the model made the model more likely.

### 5.5. Aggregation of fish

**Figure 4.**
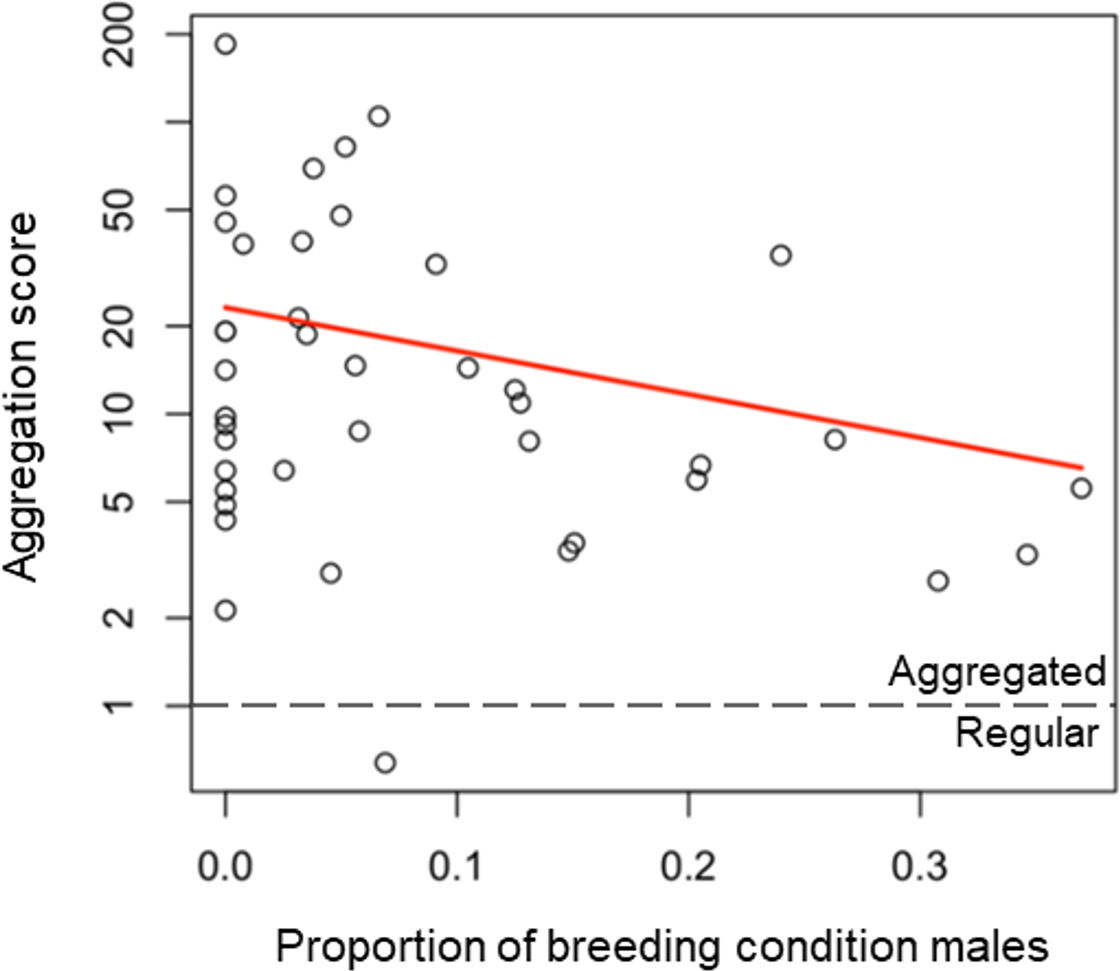
Relationship between the proportion of red-bellied males caught and the aggregation of fish. The plotted line shows the fitted relationship from GLMM coefficients. The horizontal dashed line indicates where fish are randomly distributed; below this line, fish are more evenly distributed than would occur by chance (regular distribution), and above this line, fish are more clustered than random (aggregated distribution).

Overall, the fish showed a high degree of aggregation, where more fish were caught in fewer traps than expected by chance (figure 3*a*). When testing the variables that predicted the aggregation score of fish across the traps for each sampling session, the most likely model given the data was the model with the proportion of red-bellied males as the explanatory variable (table 3). The proportion of breeding-condition males was negatively associated with the aggregation score (figure 4), i.e. fish were less aggregated when there were more breeding males (see figure 2 for a spatial illustration of these patterns). The IRR indicates a substantial decrease in the aggregation score associated with additional red males appearing in traps, where for each additional red male found within a trap, the aggregation score would decrease by 97% after controlling for other variables. All other models had larger AICc values than the null model, so were not supported by the data.

**Table 3.** The ∆AICc for models explaining the aggregation of fish. Models differ in the explanatory variable included, and all include pond site as the random effect. The null model has no explanatory variable, only the random effect. Week (weeks 1–13) and morning or afternoon (sampling in am or pm) represent when data collection occurred. The proportion of red males caught represents how many breeding-condition males were caught relative to the total number caught. The slope (coefficient), standard error of the coefficient and incidence rate ratio (IRR) for each explanatory variable are provided.

explanatory variable	slope	s.e.	IRR	∆AICc	d.f
proportion of red males caught	−3.423	1.45	0.032	0	4
null model (no explanatory variables)	NA	NA	NA	2.6	3
dissolved oxygen	0.201	0.15	1.223	3.3	4
Morning or afternoon	0.314	0.252	1.368	3.5	4
turbidity	−0.247	0.234	0.781	4	4
temperature	−0.045	0.149	1.046	4.9	4
week	0.015	0.038	1.016	4.9	4
light intensity	−0.069	0.184	0.933	5	4

### 5.6. Body size assortment of shoaling fish

**Figure 5.**
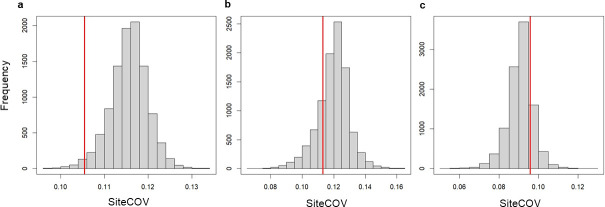
Expected distributions of the coefficient of variation (COV) of body lengths of fish caught in traps, generated by randomized sampling for three example sampling sessions. The red lines show the median of the COV for that sampling session (observed siteCOV). Quantiles, representing the proportion of expected values less than or equal to the observed value, are significant at less than 0.025 (positive assortment, fish group with similar sizes) or greater than 0.975 (negative assortment, fish group with dissimilar sizes). Examples shown include: (*a*) the only statistically significant value for positive assortment (quantile 0.004), (*b*) a non-significant value for positive assortment (quantile 0.195) and (*c*) a non-significant value for negative assortment (quantile 0.68).

We found little evidence of body length assortment among the fish caught in the traps. Out of 44 quantiles for the observed siteCOV in the distribution expected from random assortment, calculated for each sampling session, only 1 quantile was below < 0.025 (figure 5*a*). Eight sampling sessions were unsuitable for analysis due to low numbers of fish caught during the session or due to the distributions of fish across traps. The majority of sampling sessions yielded non-significant tendencies to be positively assorted (e.g. figure 5*b*); in 8 of the 44 visits, the values tended towards negative assortment (e.g. figure 5*c*), but none were statistically significant.

## 6. Discussion

Here, we present and test a novel field method for quantifying social behaviour and the impacts of environmental variables on this behaviour. We demonstrate that this method can be used with low-cost equipment in habitats where other methods for quantifying social behaviour would be unfeasible, as the pond habitats used in this study are often too turbid and/or vegetated for direct observation or filming underwater. These conditions would also make catching a large proportion of the fish population for tagging in each pond hard to achieve while incurring substantial disturbance to the fish and/or habitat.

The primary goal in developing this method is to measure social tendency in a population. We quantify social tendency using the index of dispersion as an aggregation score, as commonly used in population ecology [45]. Contributing to the validity of our approach, we find that sticklebacks were less aggregated when the proportion of red-bellied breeding-condition males caught was higher, indicating that the fish were less social during the breeding season. This is consistent with the reproductive biology of three-spined sticklebacks, where males in breeding condition maintain territories [38]. Surprisingly, we did not find that aggregation was related to any of the environmental variables we recorded, unlike laboratory studies testing a range of environmental variables on shoaling behaviour in fish [7,9,31,33]. This may be because under field conditions with variation in multiple, possibly interacting environmental parameters, the effect of any single stressor is difficult to detect in comparison to highly controlled laboratory studies [24,47]. This may also explain why the week of data collection was not related to aggregation, which was expected based on laboratory studies where repeatedly testing shoals of sticklebacks does show a reduction in aggregation over time [48]. The results of our Friedman test provided some evidence that sticklebacks have a preference for particular trap locations, indicating fish distribution is influenced by localized environmental factors (e.g. light intensity [49,50], food availability [51] and shelter [52]).

Fewer fish were caught with decreasing temperatures, at lower light levels and in areas with a higher dissolved oxygen concentration. Changes as a result of temperature can be explained by activity, because fish are ectothermic and have lower metabolisms at lower temperatures [53], resulting in reduced activity [31] and diminished exploration for food [53]. Environmental conditions may also be correlated with other parameters [54,55], for example, light intensity would be affected by canopy cover at different trap locations (canopy cover was not recorded [43,56]). Controlling for the week of data collection, we observed a decrease in the proportion of adult fish caught with increasing temperatures, likely a result of differences between adults and juveniles in size and metabolic rate [57]. Further, a higher proportion of adults were caught in the afternoon and at higher levels of light and turbidity. These results suggest that juveniles and adults respond differently depending on environmental conditions, in particular those that influence vision [58,59]. In addition to direct physiological effects, differences in activity in response to environmental variables may also arise between adults and juveniles due to trade-offs in foraging and predation risk. For example, smaller prey that are more suitable for juvenile fish may be more available earlier in the day, while predators of smaller fish may be less active [60]. It should also be noted that without directly measuring the activity of individual fish, the relative difference in the number of fish caught between adults and juveniles may be due to different responses in neophobia that vary between adults and juveniles with environmental factors [61]. Further studies are required to disentangle the underlying causes of these environmental effects on the relative capture rates of adults and juveniles in traps, and understanding group responses to environmental change more broadly [60]. Our method provides an accessible way to test hypotheses relating to the impact of environmental change on social behaviour in a natural context, however, a limitation is that we cannot directly measure what the fish are doing; here, we sacrifice finer scale information for coarser data relating to general social behaviour in the population. The benefit of this, however, is that we capture a larger proportion of the population in our data and are able to gather more data more easily.

More fish were caught in spring and summer, declining through autumn and into winter. Although variation in temperature and/or light intensity could partially explain this trend, our AICc model comparisons demonstrated that the model with the week of data collection was by far the most strongly supported model, suggesting a temporal trend in addition to those correlated with temperature and/or light intensity. This could be because the sticklebacks were learning to avoid the traps over time [62–64]. Future work could explore this further via observations of how the animals enter traps and whether they appear to actively avoid them with repeated exposure. However, it is worth noting that our approach of sampling multiple locations simultaneously in an area to measure aggregation and phenotypic assortment does not require that same area to be repeatedly sampled over time. Sampling more sites but less often per site would help minimize changes in responses to traps but would increase the among-site variation in unmeasured biotic and abiotic parameters.

A related issue and possible source of bias in our method is for certain phenotypes to be over- or under-represented due to using traps [62,65]. The effects of temperature, time of day, light intensity and turbidity on the proportion of adults caught relative to juveniles, after controlling for the week of data collection, demonstrate the potential for such bias. However, while phenotypic data such as body size or breeding condition are relatively easily measured, there is also evidence from previous work for trapping bias related to behavioural characteristics, which are much more difficult to measure. Kressler *et al.* [64] found that when using passive traps, more active fish were captured sooner, and counter-intuitively, when traps contained conspecifics, less-social fish were also captured sooner. Capture bias is something to be considered, and could even provide an interesting opportunity for further work using this method by conducting behavioural trials with fish captured by different trapping methods.

Our method is flexible in its application to different study systems when trapping techniques relevant to the habitat and the species are used. For example, camera traps could be set up for studying bird populations [28], pitfall or light traps for insect populations [66,67] or remote recording devices in large and clear bodies of water [20]. The approach can also be applied to existing datasets, where samples were taken simultaneously or can be time matched, which already have the data needed for the analysis (e.g. for calculating aggregation scores). Future applications of this method could compare sites with systemic differences, for example, polluted versus pristine environments. The biotic and abiotic components of the method can also be altered to address different hypotheses related to animal social behaviour. Biotic hypotheses could relate to phenotypic or physiological differences (e.g. colouration and parasite load) or explore effects of predation risk [4] or invasive species [68]. In the case of invasive species, the association of native and invasive species within traps could be used to infer whether these species are interacting socially or are avoiding one another [69], and whether environmental parameters affect these interactions [70].

The results from our study lend support to the validity of our method, presenting findings that we would expect in stickleback populations, but also reveal unexpected outcomes that highlight the necessity of studying wild populations. Specifically, we were able to quantify the degree to which different environmental variables influence the number of fish caught, determine whether these variables influenced aggregation (and in particular found that the proportion of breeding males in a population did reduce overall aggregation), and establish that there was no phenotypic assortment in these populations. Our method has some substantial benefits over other techniques for quantifying social behaviour in the field, particularly being cost effective and feasible, and widely applicable to a range of systems, species and hypotheses. However, careful design is needed to overcome potential problems of bias. Nevertheless, it provides an opportunity to investigate hypotheses regarding social behaviour under environmental change, anthropogenic pollution and other biotic contexts under field conditions, questions which to date have been dominated by experimental laboratory studies [7].

## Data Availability

Data and code have been archived in Dryad [71]. Supplementary material is available online [72].

## References

[1] Marras S, Killen SS, Lindström J, McKenzie DJ, Steffensen JF, Domenici P. 2015 Fish swimming in schools save energy regardless of their spatial position. Behav. Ecol. Sociobiol. **69**, 219–226. (10.1007/s00265-014-1834-4) 25620833 PMC4293471

[2] Taborsky M. 2001 The Evolution of Bourgeois, Parasitic, and Cooperative Reproductive Behaviors in Fishes. J. Hered. **92**, 100–110. (10.1093/jhered/92.2.100) 11396567

[3] Ranta E, Kaitala V. 1991 School size affects individual feeding success in three‐spined sticklebacks (Gastevosteus aculeatus L.). J. Fish Biol. **39**, 733–737. (10.1111/j.1095-8649.1991.tb04402.x)

[4] Ioannou C. 2021 Grouping and Predation. In Encyclopedia of evolutionary psychological science (eds TK Shackelford, VA Weekes-Shackelford), pp. 3574–3580. Cham, Switzerland: Springer. (10.1007/978-3-319-19650-3_2699)

[5] Grand TC Dill LM 1999 The effect of group size on the foraging behaviour of juvenile coho salmon: reduction of predation risk or increased competition? Anim. Behav. **58**, 443–451. (10.1006/anbe.1999.1174) 10458896

[6] Krause J, Ruxton GD. 2002 Living in groups. Online edition, Oxford Academic: Oxford. (10.1093/oso/9780198508175.001.0001)

[7] Fisher DN, Kilgour RJ, Siracusa ER, Foote JR, Hobson EA, Montiglio PO, Saltz JB, Wey TW, Wice EW. 2021 Anticipated effects of abiotic environmental change on intraspecific social interactions. Biol. Rev. **96**, 2661–2693. (10.1111/brv.12772) 34212487

[8] Michelangeli M, Martin JM, Pinter-Wollman N, Ioannou CC, McCallum ES, Bertram MG, Brodin T. 2022 Predicting the impacts of chemical pollutants on animal groups. Trends Ecol. Evol. **37**, 789–802. (10.1016/j.tree.2022.05.009) 35718586

[9] MacGregor HEA, Ioannou CC. 2023 Shoaling behaviour in response to turbidity in three‐spined sticklebacks. Ecol. Evol. **13**, e10708. (10.1002/ece3.10708) 37941736 PMC10630046

[10] Ginnaw GM, Davidson IK, Harding HR, Simpson SD, Roberts NW, Radford AN, Ioannou CC. 2020 Effects of multiple stressors on fish shoal collective motion are independent and vary with shoaling metric. Anim. Behav. **168**, 7–17. (10.1016/j.anbehav.2020.07.024)

[11] Blumstein DT, Petelle MB, Wey TW. 2013 Defensive and social aggression: repeatable but independent. Behav. Ecol. **24**, 457–461. (10.1093/beheco/ars183)

[12] Teichroeb JA, White MMJ, Chapman CA. 2015 Vervet (Chlorocebus pygerythrus) Intragroup Spatial Positioning: Dominants Trade-Off Predation Risk for Increased Food Acquisition. Int. J. Primatol. **36**, 154–176. (10.1007/s10764-015-9818-4)

[13] Doran C *et al*. 2022 Fish waves as emergent collective antipredator behavior. Curr. Biol. **32**, 708–714.(10.1016/j.cub.2021.11.068) 34942081

[14] Ginelli F, Peruani F, Pillot MH, Chaté H, Theraulaz G, Bon R. 2015 Intermittent collective dynamics emerge from conflicting imperatives in sheep herds. Proc. Natl Acad. Sci. USA **112**, 12729–12734. (10.1073/pnas.1503749112) 26417082 PMC4611628

[15] Nagy M, Akos Z, Biro D, Vicsek T. 2010 Hierarchical group dynamics in pigeon flocks. Nature **464**, 890–893. (10.1038/nature08891) 20376149

[16] Aplin LM, Farine DR, Morand-Ferron J, Sheldon BC. 2012 Social networks predict patch discovery in a wild population of songbirds. Proc. R. Soc. B **279**, 4199–4205. (10.1098/rspb.2012.1591) PMC3441092 22915668

[17] Savill KL, Parnum I, McIlwain J, Belton D. Influence of camera type, height and image enhancement on photogrammetry success in turbid marine environments. bioRxiv (10.1101/2024.09.15.613158)

[18] Smukall MJ, Kessel ST, Franks BR, Feldheim KA, Guttridge TL, Gruber SH. 2019 No apparent negative tagging effects after 13 years at liberty for lemon shark, Negaprion brevirostris implanted with acoustic transmitter. J. Fish Biol. **94**, 173–177. (10.1111/jfb.13856) 30393865

[19] Macaulay G, Warren‐Myers F, Barrett LT, Oppedal F, Føre M, Dempster T. 2021 Tag use to monitor fish behaviour in aquaculture: a review of benefits, problems and solutions. Rev. Aquac. **13**, 1565–1582. (10.1111/raq.12534)

[20] Sarà G *et al*. 2007 Effect of boat noise on the behaviour of bluefin tuna Thunnus thynnus in the Mediterranean Sea. Mar. Ecol. Prog. Ser. **331**, 243–253. (10.3354/meps331243)

[21] Szopa-Comley AW, Duffield C, Ramnarine IW, Ioannou CC. 2020 Predatory behaviour as a personality trait in a wild fish population. Anim. Behav. **170**, 51–64. (10.1016/j.anbehav.2020.10.002)

[22] Taylor IG, Gertseva V, Methot RD Jr, Maunder MN. 2013 A stock–recruitment relationship based on pre-recruit survival, illustrated with application to spiny dogfish shark. Fish. Res. **142**, 15–21. (10.1016/j.fishres.2012.04.018)

[23] Kilfoil JP, Campbell MD, Heithaus MR, Zhang Y. 2021 The influence of shark behavior and environmental conditions on baited remote underwater video survey results. Ecol. Model. **447**, 109507. (10.1016/j.ecolmodel.2021.109507)

[24] Ormerod SJ, Dobson M, Hildrew AG, Townsend CR. 2010 Multiple stressors in freshwater ecosystems. Freshw. Biol. **55**, 1–4. (10.1111/j.1365-2427.2009.02395.x)

[25] Castello L, McGrath DG, Hess LL, Coe MT, Lefebvre PA, Petry P, Macedo MN, Renó VF, Arantes CC. 2013 The vulnerability of Amazon freshwater ecosystems. Conserv. Lett. **6**, 217–229. (10.1111/conl.12008)

[26] Handegard NO, Boswell KM, Ioannou CC, Leblanc SP, Tjøstheim DB, Couzin ID. 2012 The Dynamics of Coordinated Group Hunting and Collective Information Transfer among Schooling Prey. Curr. Biol. **22**, 1213–1217. (10.1016/j.cub.2012.04.050) 22683262

[27] Rodriguez-Pinto II, Rieucau G, Handegard NO, Boswell KM. 2020 Environmental context elicits behavioural modification of collective state in schooling fish. Anim. Behav. **165**, 107–116. (10.1016/j.anbehav.2020.05.002)

[28] O’Brien TG, Kinnaird MF. 2008 A picture is worth a thousand words: the application of camera trapping to the study of birds. Bird Conserv. Int. **18**, S144–S162. (10.1017/s0959270908000348)

[29] Gartland LA, Firth JA, Laskowski KL, Jeanson R, Ioannou CC. 2022 Sociability as a personality trait in animals: methods, causes and consequences. Biol. Rev. Camb. Philos. Soc. **97**, 802–816. (10.1111/brv.12823) 34894041

[30] Ranta E, Lindström K. 1990 Assortative schooling in three-spined sticklebacks? Ann. Zool. Fenn. **27**, 67–75.

[31] Bartolini T, Butail S, Porfiri M. 2015 Temperature influences sociality and activity of freshwater fish. Environ. Biol. Fishes **98**, 825–832. (10.1007/s10641-014-0318-8)

[32] Zanghi C, Munro M, Ioannou CC. 2023 Temperature and turbidity interact synergistically to alter anti-predator behaviour in the Trinidadian guppy. Proc. R. Soc. B **290**, 20230961. (10.1098/rspb.2023.0961) PMC10320334 37403508

[33] Domenici P, Ferrari RS, Steffensen JF, Batty RS. 2002 The effect of progressive hypoxia on school structure and dynamics in Atlantic herring Clupea harengus. Proc. R. Soc. B **269**, 2103–2111. (10.1098/rspb.2002.2107) PMC1691132 12396484

[34] Ohata R, Masuda R, Takahashi K, Yamashita Y. 2014 Moderate turbidity enhances schooling behaviour in fish larvae in coastal waters. ICES J. Mar. Sci. **71**, 925–929. (10.1093/icesjms/fss194)

[35] Pitcher TJ, Turner JR. 1986 Danger at dawn: experimental support for the twilight hypothesis in shoaling minnows. J. Fish Biol. **29**, 59–70. (10.1111/j.1095-8649.1986.tb04999.x)

[36] Ioannou CC, Couzin ID, James R, Croft DP, Krause J. 2011 Social organisation and information transfer in schooling fish. In Fish cognition and behaviour, pp. 217–239, 2nd edn. Hoboken, New Jersey, USA: Wiley-Blackwell. (10.1002/9781444342536.ch10)

[37] R Core Team. 2017 R: A language and environment for statistical computing. R Foundation for Statistical Computing. Vienna, Austria. See https://www.R-project.org/.

[38] Tinbergen N. 1952 The Curious Behavior of the Stickleback. Sci. Am. **187**, 22–26. (10.1038/scientificamerican1252-22)

[39] Schneider CA, Rasband WS, Eliceiri KW. 2012 NIH Image to ImageJ: 25 years of image analysis. Nat. Methods. **9**, 671–675. (10.1038/nmeth.2089) 22930834 PMC5554542

[40] Brooks ME, Kristensen K, van Benthem K, Magnusson A, Berg CW, Nielsen A, Skaug HJ, Mächler M, Bolker BM. 2017 glmmTMB Balances Speed and Flexibility Among Packages for Zero-inflated Generalized Linear Mixed Modeling. R. J. **9**, 378. (10.32614/RJ-2017-066)

[41] Hartig F. 2019 DHARMa: Residual Diagnostics for Hierarchical (Multi-Level / Mixed) Regression Models.

[42] Burnham KP, Anderson DR. 1998 Model selection and inference: a practical information-theoretic approach, pp. 75–117, 2nd edn. New York, NY: Springer. (10.1007/978-1-4757-2917-7_3)

[43] Zanghi C, Penry-Williams IL, Genner MJ, Deacon AE, Ioannou CC. 2024 Multiple environmental stressors affect predation pressure in a tropical freshwater system. Commun. Biol. **7**. (10.1038/s42003-024-06364-6) PMC11137014 38811776

[44] Bolker B, Team RDC, Giné-Vázquez I. 2007 bbmle: Tools for General Maximum Likelihood Estimation (1.0.25) https://CRAN.R-project.org/package=bbmle

[45] Perry JN, Hewitt M. 1991 A New Index of Aggregation for Animal Counts. Biometrics **47**, 1505. (10.2307/2532402) 1786328

[46] Croft DP, Darden SK, Ruxton GD. 2009 Predation risk as a driving force for phenotypic assortment: a cross-population comparison. Proc. R. Soc. B **276**, 1899–1904. (10.1098/rspb.2008.1928) PMC2674500 19324770

[47] Côté IM, Darling ES, Brown CJ. 2016 Interactions among ecosystem stressors and their importance in conservation. Proc. R. Soc. B Biol. Sci. **283**, 20152592. (10.1098/rspb.2015.2592) PMC4760168 26865306

[48] MacGregor HEA, Ioannou CC. 2021 Collective motion diminishes, but variation between groups emerges, through time in fish shoals. R. Soc. Open Sci. **8**, 210655. (10.1098/rsos.210655) 34703618 PMC8527212

[49] Ryer CH, Olla BL. 1998 Effect of light on juvenile walleye pollock shoaling and their interaction with predators. Mar. Ecol. Prog. Ser. **167**, 215–226. (10.3354/meps167215)

[50] Xue T, Li X, Lin G, Escobedo R, Han Z, Chen X, Sire C, Theraulaz G. 2023 Tuning social interactions’ strength drives collective response to light intensity in schooling fish. PLoS Comput. Biol. **19**, e1011636. (10.1371/journal.pcbi.1011636) 37976299 PMC10691717

[51] Macreadie PI, Hindell JS, Keough MJ, Jenkins GP, Connolly RM. 2010 Resource distribution influences positive edge effects in a seagrass fish. Ecology **91**, 2013–2021. (10.1890/08-1890.1) 20715624

[52] Chrétien E, Boisclair D, Cooke SJ, Killen SS. 2021 Social Group Size and Shelter Availability Influence Individual Metabolic Traits in a Social Fish. Integr. Org. Biol. **3**, obab032. (10.1093/iob/obab032) 34859193 PMC8633746

[53] Clarke A, Johnston NM. 1999 Scaling of metabolic rate with body mass and temperature in teleost fish. J. Anim. Ecol. **68**, 893–905. (10.1046/j.1365-2656.1999.00337.x) 20180875

[54] Rydberg L, Edler L, Floderus S, Graneli W. 1990 Interaction between supply of nutrients, primary production, sedimentation and oxygen consumption in SE Kattegat. AMBIO. J. Hum. Environ. **19**, 134–141. (10.1016/j.seares.2006.03.009)

[55] Hagy JD, Boynton WR, Keefe CW, Wood KV. 2004 Hypoxia in Chesapeake Bay, 1950–2001: Long-term change in relation to nutrient loading and river flow. Estuaries **27**, 634–658. (10.1007/bf02907650)

[56] Ilha P, Schiesari L, Yanagawa FI, Jankowski K, Navas CA. 2018 Deforestation and stream warming affect body size of Amazonian fishes. PLoS One **13**, e0196560. (10.1371/journal.pone.0196560) 29718960 PMC5931656

[57] Garenc C, Couture P, Laflamme MA, Guderley H. 1999 Metabolic correlates of burst swimming capacity of juvenile and adult threespine stickleback (Gasterosteus aculeatus). J. Comp. Physiol. B **169**, 113–122. (10.1007/s003600050201)

[58] Evans BI, Fernald RD. 1990 Metamorphosis and fish vision. J. Neurobiol. **21**, 1037–1052. (10.1002/neu.480210709) 2258720

[59] Cortesi F, Musilová Z, Stieb SM, Hart NS, Siebeck UE, Cheney KL, Salzburger W, Marshall NJ. 2016 From crypsis to mimicry: changes in colour and the configuration of the visual system during ontogenetic habitat transitions in a coral reef fish. J. Exp. Biol. **219**, 2545–2558. (10.1242/jeb.139501) 27307489

[60] Pilakouta N *et al*. 2023 A warmer environment can reduce sociability in an ectotherm. Glob. Chang. Biol. **29**, 206–214. (10.1111/gcb.16451) 36259414 PMC10092372

[61] Bell AM, Stamps JA. 2004 Development of behavioural differences between individuals and populations of sticklebacks, Gasterosteus aculeatus. Anim. Behav. **68**, 1339–1348. (10.1016/j.anbehav.2004.05.007)

[62] Wilson DS, Coleman K, Clark AB, Biederman L. 1993 Shy-bold continuum in pumpkinseed sunfish (Lepomis gibbosus): An ecological study of a psychological trait. J. Comp. Psychol. **107**, 250–260. (10.1037/0735-7036.107.3.250)

[63] Michelangeli M, Wong BBM, Chapple DG. 2016 It’s a trap: sampling bias due to animal personality is not always inevitable. Behav. Ecol. **27**, 62–67. (10.1093/beheco/arv123)

[64] Kressler MM, Gerlam A, Spence‐Jones H, Webster MM. 2021 Passive traps and sampling bias: Social effects and personality affect trap entry by sticklebacks. Ethology **127**, 446–452. (10.1111/eth.13148)

[65] Biro PA, Dingemanse NJ. 2009 Sampling bias resulting from animal personality. Trends Ecol. Evol. **24**, 66–67. (10.1016/j.tree.2008.11.001) 19110338

[66] Jonason D, Franzén M, Ranius T. 2014 Surveying Moths Using Light Traps: Effects of Weather and Time of Year. PLoS One **9**, e92453. (10.1371/journal.pone.0092453) 24637926 PMC3956935

[67] Brown GR, Matthews IM. 2016 A review of extensive variation in the design of pitfall traps and a proposal for a standard pitfall trap design for monitoring ground‐active arthropod biodiversity. Ecol. Evol. **6**, 3953–3964. (10.1002/ece3.2176) 27247760 PMC4867678

[68] Strayer DL, Eviner VT, Jeschke JM, Pace ML. 2006 Understanding the long-term effects of species invasions. Trends Ecol. Evol. **21**, 645–651. (10.1016/j.tree.2006.07.007) 16859805

[69] Camacho-Cervantes M, Garcia CM, Ojanguren AF, Magurran AE. 2014 Exotic invaders gain foraging benefits by shoaling with native fish. R. Soc. Open Sci. **1**, 140101. (10.1098/rsos.140101) 26064552 PMC4448845

[70] Glotzbecker GJ, Ward JL, Walters DM, Blum MJ. 2015 Turbidity alters pre‐mating social interactions between native and invasive stream fishes. Freshw. Biol. **60**, 1784–1793. (10.1111/fwb.12610)

[71] Clark MA, Ioannou CC. 2024 Quantifying animal social behaviour with ecological field methods [Dataset]. Dryad (10.5061/dryad.g79cnp5zw) PMC11732413 39816731

[72] Clark MA, Ioannou CC. 2024 Supplementary material from: Quantifying animal social behaviour with ecological field methods. Figshare. (10.6084/m9.figshare.c.7585460) PMC11732413 39816731

